# Embryonic POU5F1 is Required for Expanded Bovine Blastocyst Formation

**DOI:** 10.1038/s41598-018-25964-x

**Published:** 2018-05-17

**Authors:** Bradford W. Daigneault, Sandeep Rajput, George W. Smith, Pablo J. Ross

**Affiliations:** 1 Department of Animal Science, Michigan State University, East-Lansing, Michigan USA; 2 Department of Animal Science, University of California Davis, Davis, CA USA

## Abstract

POU5F1 is a transcription factor and master regulator of cell pluripotency with indispensable roles in early embryo development and cell lineage specification. The role of embryonic POU5F1 in blastocyst formation and cell lineage specification differs between mammalian species but remains completely unknown in cattle. The CRISPR/Cas9 system was utilized for targeted disruption of the *POU5F1* gene by direct injection into zygotes. Disruption of the bovine *POU5F1* locus prevented blastocyst formation and was associated with embryonic arrest at the morula stage. POU5F1 knockout morulas developed at a similar rate as control embryos and presented a similar number of blastomeres by day 5 of development. Initiation of SOX2 expression by day 5 of development was not affected by lack of POU5F1. On the other hand, CDX2 expression was aberrant in embryos lacking POU5F1. Notably, the phenotype observed in bovine *POU5F1* knockout embryos reveals conserved functions associated with loss of human embryonic POU5F1 that differ from *Pou5f1*- null mice. The similarity observed in transcriptional regulation of early embryo development between cattle and humans combined with highly efficient gene editing techniques make the bovine a valuable model for human embryo biology with expanded applications in agriculture and assisted reproductive technologies.

## Introduction

POU5F1 is a transcription factor and developmental control gene with regulatory roles in mammalian embryo development, cell lineage specification and maintenance of germ cell pluripotency^1^. POU5F1 functions as a homeodomain transcription factor of the POU (Pit-Oct-Unc) family by binding to a specific octameric sequence motif (ATGCAAT) on enhancer and promoter regions of target genes through a POU domain to activate and repress gene expression^2,3^. Although POU5F1 is well-recognized for many roles in mammalian embryo development^2^, the requirement of embryonic POU5F1 in bovine blastocyst formation and cell lineage specification has not been determined in fertilized embryos. Technical imitations with gene targeting and the long generational interval of livestock species have accounted for the inability to address distinct biological roles of developmental control genes in cattle. In addition, prior to the adoption of CRISPR/Cas9 for use in livestock, defining roles for genomic regulation of bovine blastocyst formation was confounded by the inability to discriminate the functions of maternal and embryonic transcripts with loss of function approaches^4^. However, characterization studies of bovine POU5F1 indicate an important biological role in embryo development^5–9^ with embryonic POU5F1 expression beginning at the 8–16 cell stage^6^. The function of maternal POU5F1 transcripts has not been differentiated from embryonic POU5F1 using traditional siRNA-mediated knockdown approaches for functional studies of embryonic genes in bovine embryo development^9^. Direct zygote injection of CRISPR/Cas9 components targeting developmental control genes represents a powerful tool to accurately determine gene function^4,10^.

Mouse models have provided insight regarding the roles of POU5F1 during mammalian preimplantation development^11^ yet major species differences in the timing of gene activation, spatio-temporal expression and regulatory interactions of POU5F1 with other transcription factors suggest differences in the requirement of POU5F1 for cattle and mice^12–14^. In contrast, similarities in spatio-temporal gene expression and gastrulation of bovine and human embryo development may indicate conserved roles for early developmental genes such as *POU5F1* that could provide a valuable model for understanding human developmental biology and development of assisted reproductive technologies^13^. Among mammals, POU5F1 is consistently expressed at EGA, suggesting some conserved roles in early development, including cell lineage specification and blastocyst formation^2^. Contrary to the mouse, bovine POU5F1 is not restricted to the inner-cell mass (ICM) but is ubiquitous to both the ICM and trophectoderm (TE) lineages of the early blastocyst^6,7,15^. In pre-compaction mouse embryos, POU5F1 is ubiquitous and becomes restricted to the ICM during the first cell lineage specification^16,17^. CDX2 is a transcription factor specifically expressed in the TE lineage and is associated with downregulation of POU5F1 in mouse embryos, while in bovine POU5F1 is not immediately repressed by TE formation^17,18^. Depletion of *Pou5f1* in mouse embryos does not prevent blastocyst formation or establishment of the epiblast (EPI) and TE lineages, but is required for proper development of the primitive endoderm (PE) as well as expression of multiple EPI and PE genes, such as GATA6 and FGF4^11^. Recent evidence obtained from human embryos suggests an earlier requirement for POU5F1 with biologically different roles than those described for mouse embryos, such as primary cell lineage specification and blastocyst formation^19^.

We hypothesized that bovine POU5F1 plays an important role in bovine embryo development distinct from rodents due to differences in the timing of expression, localization and regulatory interactions. The requirement of embryonic POU5F1 in bovine blastocyst development and cell lineage specification was investigated using a loss-of-function approach based on direct zygotic injection of CRISPR/Cas9 components.

## Results

### CRISPR-induced *POU5F1* mutations prevent blastocyst formation in bovine embryos

**Figure 1:**
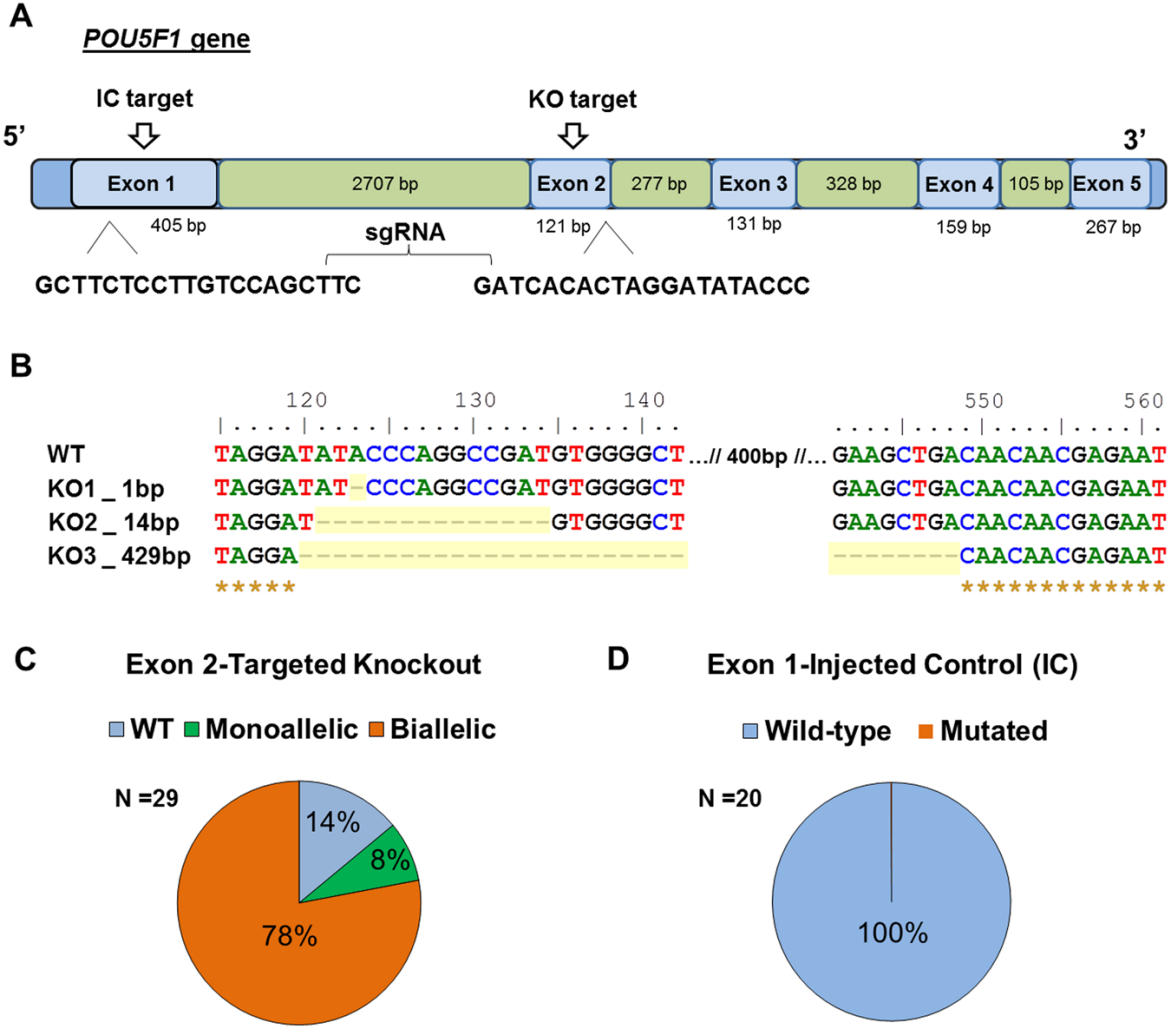
Efficiency of CRISPR-Cas9 *POU5F1* targeted deletion in bovine embryos. (**A**) Target sites and sgRNA sequences designed to disrupt the coding regions of bovine *POU5F1*. (**B**) Representation of indel mutations as a result of intracytoplasmic zygotic injection of Cas9 and sgRNA targeting exon 2 of *POU5F1*. (**C**) *POU5F1* mutation efficiency for exon 2 observed in D3 (8–16C) morulas. (**D**) Mutation efficiency in D3 (8–16C) embryos when targeting exon 1 of *POU5F1*.

A synthetic guide RNA targeting exon 2 (E2) of bovine *POU5F1* was microinjected into the cytoplasm of bovine zygotes in complex with Cas9 protein (Fig. 1A). Targeted mutation of embryonic *POU5F1* E2 was highly efficient, with large deletions up to 429 bp in length (Fig. 1B), resulting in a total KO rate of 86% (n = 29, 8–16C embryos; Fig. 1C). Furthermore, 78% of sequenced embryos targeted for knockout (TKO) yielded bi-allelic mutations. The mutation efficiency when targeting exon1 (E1) of embryonic *POU5F1* yielded wild-type embryos only and thus served as an injected control (IC) for further experiments (Fig. 1D).

**Figure 2:**
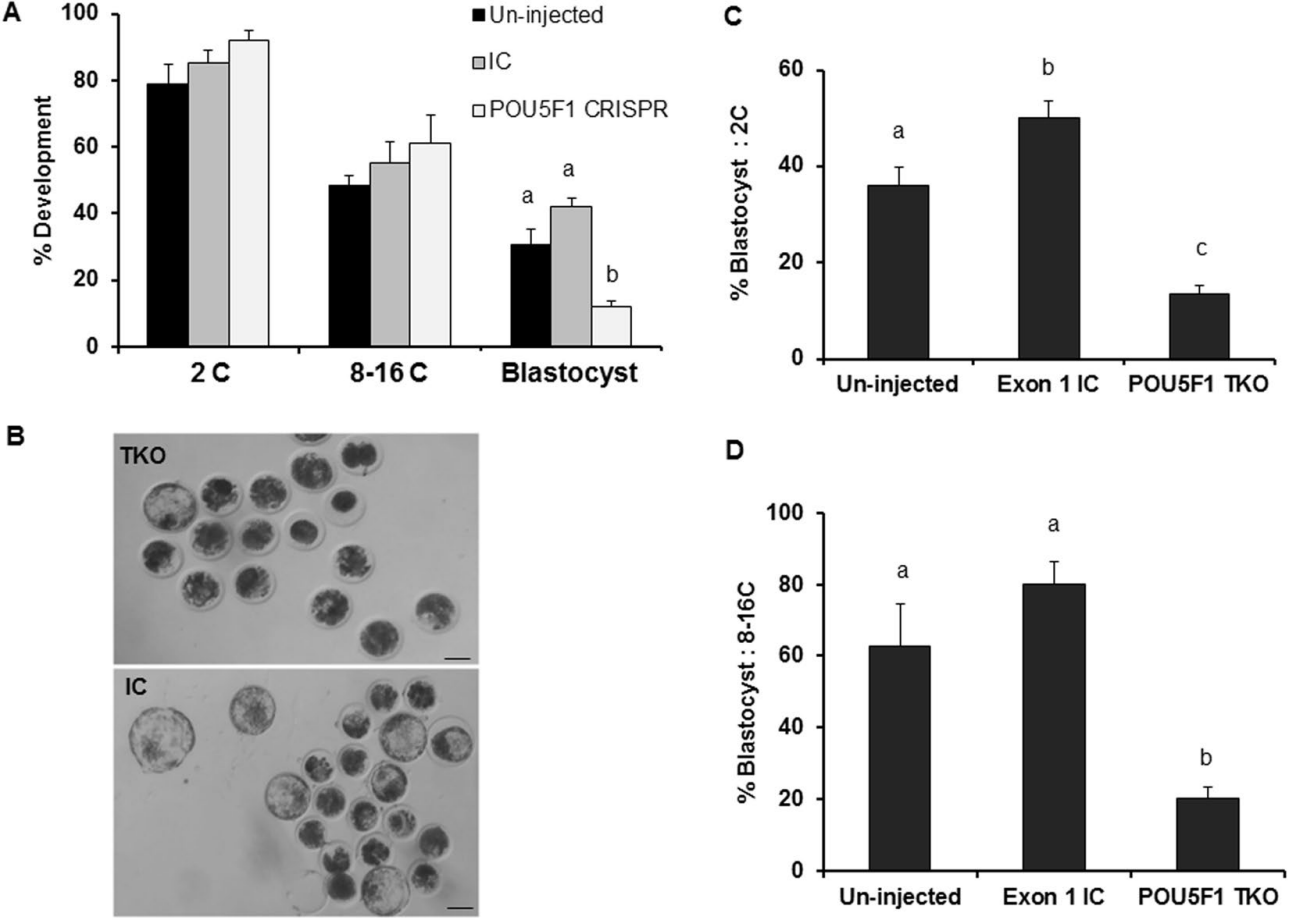
Effect of *POU5F1* CRISPR-induced mutation on bovine embryo development. (**A**) Embryo development following zygotic injection of CRISPR/Cas9 targeting the *POU5F1* locus was determined at the 2C (cell), 8–16C and blastocyst stages (D7.5). (**B**) Representative brightfield image of D7.5 embryo morphology and blastocyst development in targeted knockout and injected controls. (**C**,**D**) Blastocyst development calculated based on the number of embryos reaching the 2C and 8–16 stage, respectively. ^a,b^Different letters indicate significant differences (P < 0.05). Scale bar = 100 µm.

The requirement of embryonic *POU5F1* for bovine preimplantation development was tested by evaluating the developmental potential of embryos targeted for *POU5F1* mutations. No differences in embryo cleavage (2C) and development to the 8–16C (cell) stage were observed between targeted and control embryos (Fig. 2A). Blastocyst formation in the TKO group was significantly lower (P < 0.05) compared to injected and un-injected controls (12, 42 and 31%, respectively), with no differences in blastocyst development among controls (P > 0.05, Figure A,B). Similarly, the proportion of 2C and 8C embryos developing to blastocyst stage was lower in TKO group compared to controls (P < 0.05; Fig. 2C,D).

**Figure 3:**
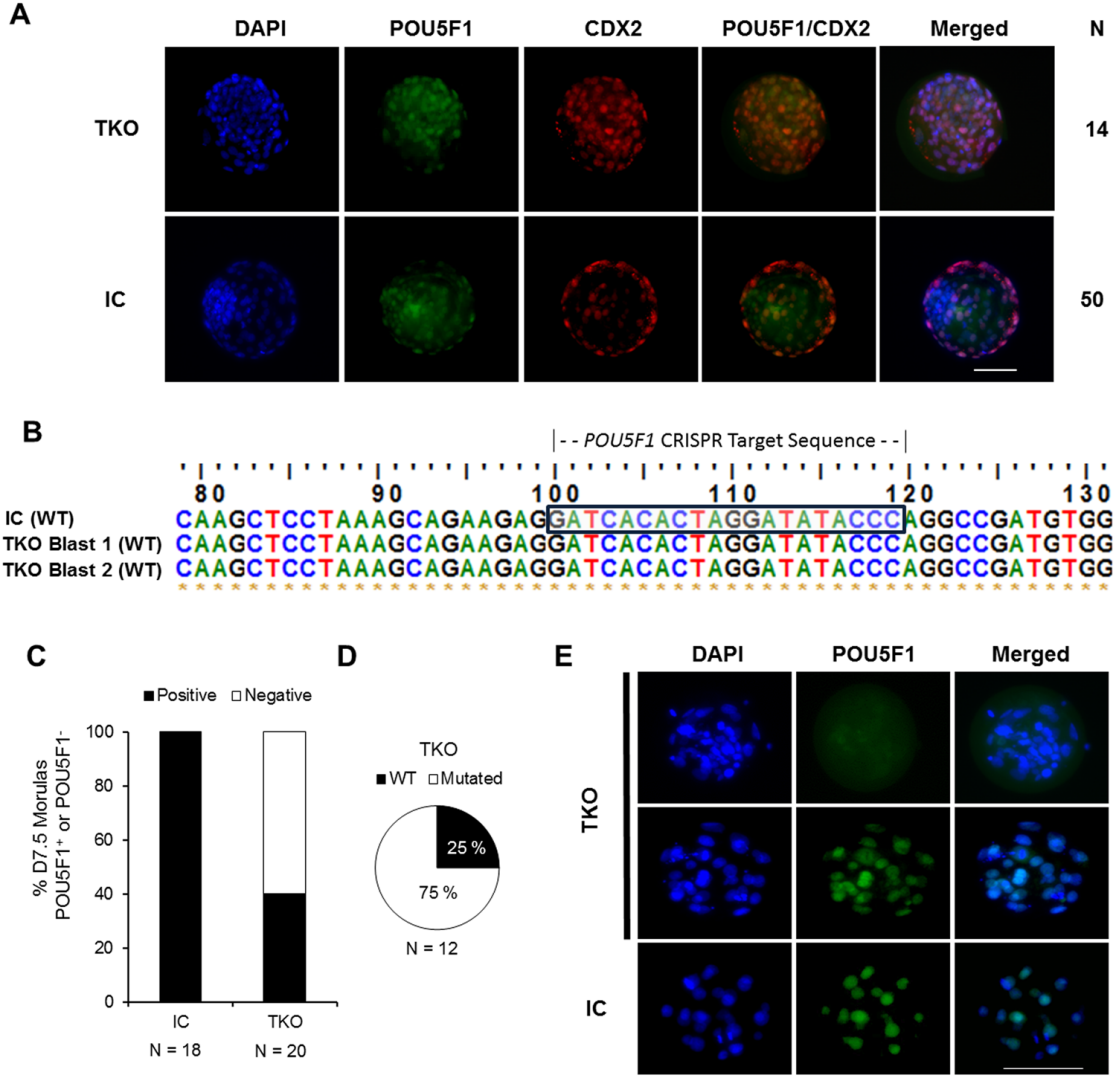
POU5F1 expression is required for blastocyst formation in bovine embryos. (**A**) Immunofluorescence analysis of POU5F1 (green) and CDX2 (red) expression in D7.5 expanded blastocysts from the POU5F1 targeted knockout and injected control (IC) groups. (**B**) Genotype analyses from D7.5 blastocysts (n = 2) following confirmation of POU5F1 expression in embryos targeted for knockout. (C) Proportion of morulas collected on D7.5 that were stained and determined as POU5F1 positive (+/+, +/−) or negative (−/−). (**D**) Embryos in the targeted knockout group that failed to form blastocysts by D7.5 were genotyped to determine total mutation rate and allelic knockout efficiency (bi-alleic or monoallelic). (**E**) Immunofluorescence analyses of POU5F1 (green) and DAPI (blue) staining in D7.5 arrested morulas from targeted knockout and injected control groups. Scale bar = 100 µm.

Day 7.5 embryos that reached the expanded blastocyst stage following zygotic injection were subjected to immunostaining to determine relative expression of POU5F1and CDX2 in both TKO and IC groups. All (n = 14) expanded blastocysts in the TKO group (n = 5 reps) stained positive for POU5F1 and CDX2 and displayed similar POU5F1 ubiquitous staining patterns compared to injected controls (Fig. 3A), indicating that embryos developing to blastocyst stage contained a functional copy of the *POU5F1* gene. To confirm their genotype, two embryos from the TKO group, that were positive for POU5F1 staining, were sequenced following immunofluorescence analysis and were determined to be wild-type embryos (Fig. 3B).

Sequence analysis of D7.5 arrested embryos revealed a 75% *POU5F1* mutation rate within the TKO group (Fig. 3C,D). POU5F1 protein was detected in 40% of TKO D7.5 arrested/delayed embryos while it was observed in 100% of injected controls (Fig. 3C). When positive, POU5F1 staining appeared similar in TKO embryos compared to injected controls (Fig. 3E).

### CDX2 is aberrantly expressed in D7.5 arrested morulas

**Figure 4:**
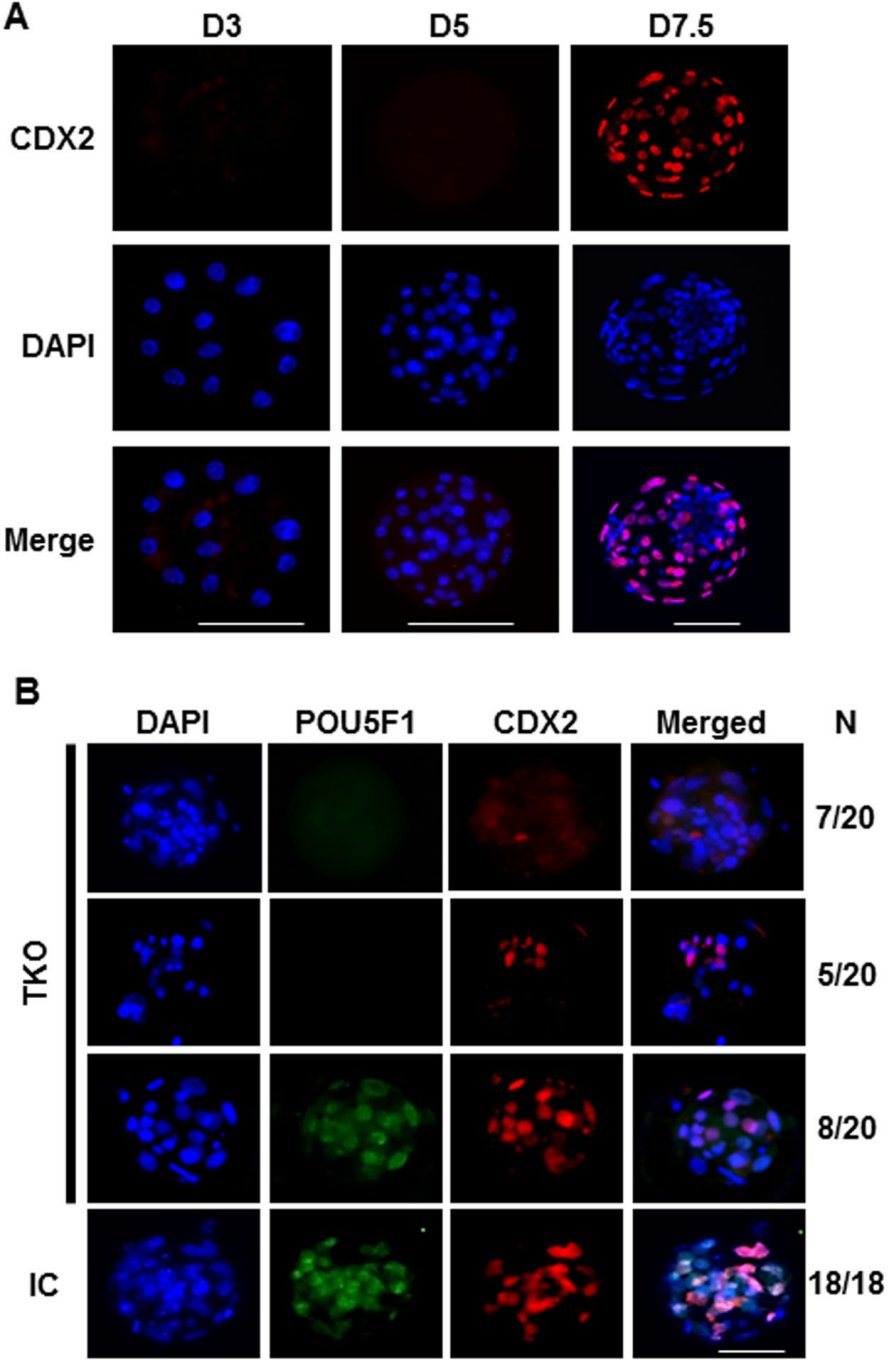
Failure to reach blastocyst in *POU5F1* knockout embryos is associated with altered CDX2 expression. (**A**) Characterization of CDX2 (red) expression in D3, 5 and 7.5 morulas from injected control groups. (**B**) CDX2 (red) staining patterns detected in D7.5 arrested POU5F1-edited and unedited embryos from the targeted knockout and injected control groups. Scale bar = 100 µm.

CDX2 expression was evaluated in IC embryos at D3, 5 and 7.5 to determine the timing of trophectoderm lineage specification. CDX2 was absent at D3 and D5 but was strongly expressed in D7.5 blastocysts and morulas (Fig. 4A). The expression of CDX2 was then determined in association with POU5F1 expression in D7.5 arrested morulas (Fig. 4B). In TKO D7.5 morulas that expressed POU5F1, CDX2 staining was similar to controls. However, TKO D7.5 morulas lacking POU5F1 expression presented a weak and inconsistent CDX2 staining (Fig. 4B).

### POU5F1 knockout allows normal development to D5 morula stage

**Figure 5:**
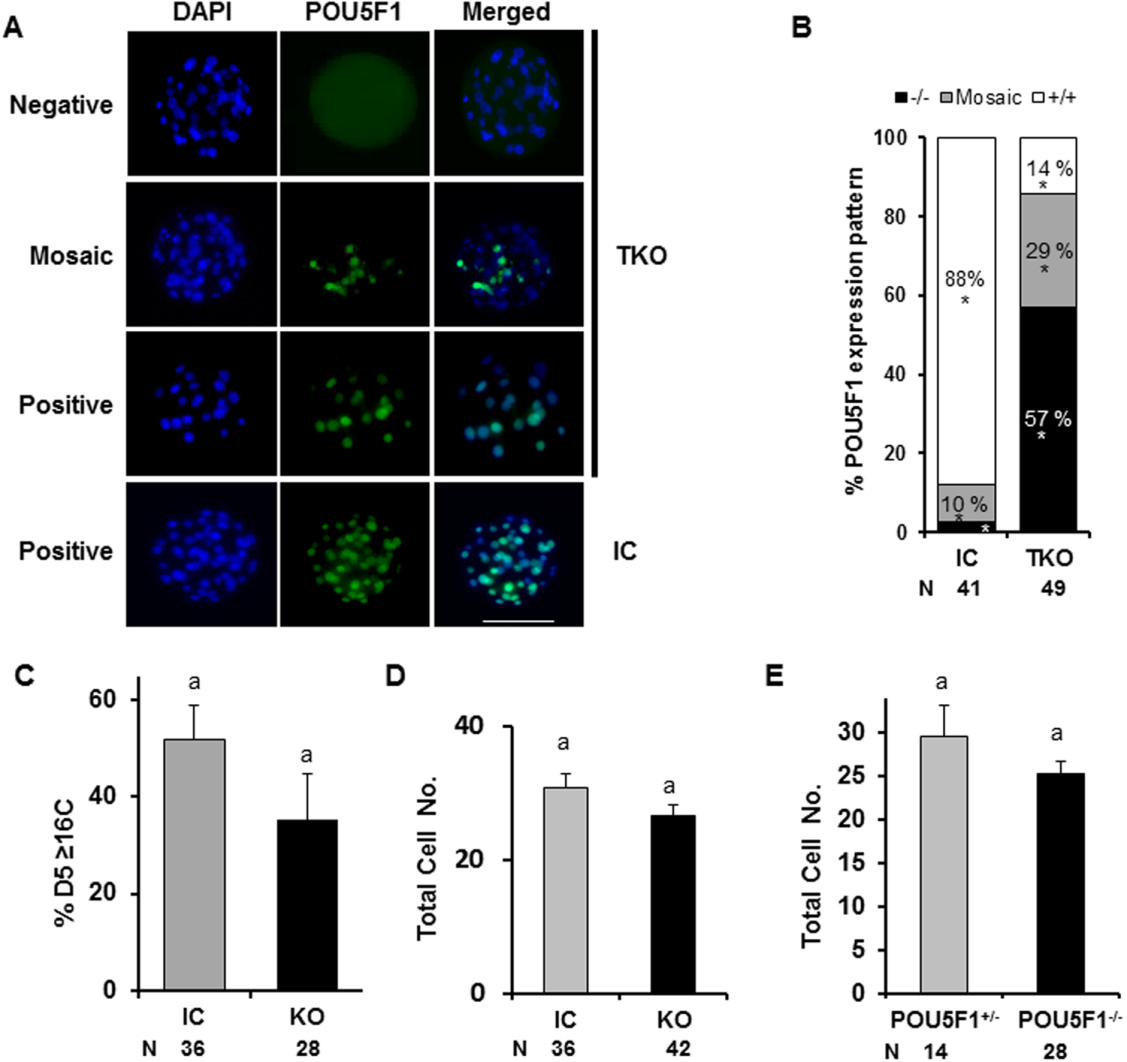
Embryonic POU5F1 is not required for D5 morula development. (**A**) Immunofluorescence staining for POU5F1 revealed embryos with negative, mosaic (MO) and positive expression in the CRISPR-targeted knockout group. (**B**) Quantification and characterization of POU5F1 expression patterns as negative (−/−), Mosaic or positive (+/+: >75% positive cells) from injected control and POU5F1 targeted embryos. * indicates difference (P < 0.05) between groups (TKO vs. IC) within mutation type. (**C**) Percentage of morulas with ≥16C that advanced from D3 to D5 in control and POU5F1 targeted groups. (**D**) Total cell number from injected control and targeted knockout (+/−, −/−) embryos. (**E**) Total cell number in targeted knockout embryos with zero (POU5F1^−/−^) or mosaic (POU5F1^+/−^) POU5F1 staining.

The POU5F1 knockout embryos did not differ in their ability to reach the 8–16C stage compared to controls, but underwent developmental arrest at morula stage (Fig. 3). To assess the phenotypic characteristics of embryos at the time of arrest, D5 morulas were evaluated. Embryos targeted for deletion were discarded if 8–16C development was not achieved by 72 h post-activation and remaining embryos cultured until D5, at which time morulas were fixed and immunostained to detect POU5F1 protein (Fig. 5). When D5 embryos were evaluated, POU5F1 was detected in all the cells from control embryos, while in TKO embryos, three different phenotypes were observed. Some embryos showed all cells stained with POU5F1, some were completely negative for POU5F1 expression, and some were mosaic, with some cells staining positive and others staining negative. This last group is consistent with mutation chimerisms observed after CRISPR/Cas9 direct injection into zygotes attributed to gene editing following DNA replication^20^ (Fig. 5A). Only 14% of embryos in the TKO group had ubiquitous (>75% of the cells) POU5F1 expression compared to 88% in the IC group (P < 0.05) as determined by immunostaining (Fig. 5B). Advancement from D3–5 was not altered (P > 0.05) in IC (70%) vs. TKO (59%) groups for embryos containing ≥16 cells (Fig. 5C). Total cell number was similar (P > 0.05) for D5 IC or TKO embryos (Fig. 5D) and also not different (P > 0.05) for TKO embryos lacking POU5F1 expression (Fig. 5E).

### SOX2 expression is not altered in POU5F1 knockout embryos

**Figure 6:**
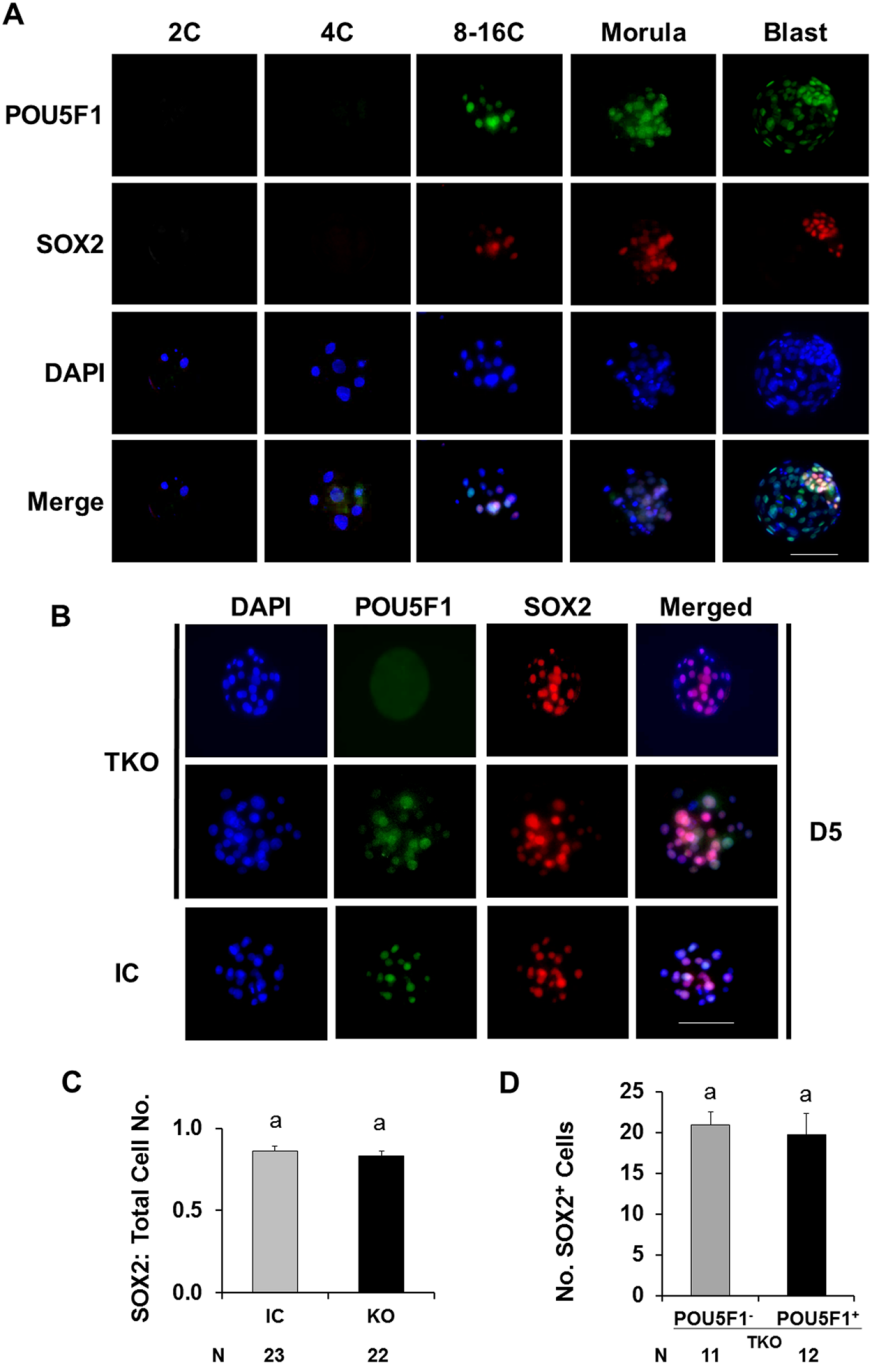
Lack of *POU5F1* does not alter SOX2 expression in D5 bovine morulas. (**A**) Temporal expression of SOX2 (red) and POU5F1 (green) in bovine embryos. (**B**) SOX2 (red) expression in POU5F1 (green) knockout and injected control D5 morulas. (**C**) Ratio of SOX2 positive cells to total cell number from injected control and POU5F1 targeted knockout groups (**D**). Number of SOX2 positive cells in POU5F1 positive and negative embryos within the targeted knockout group. Scale bar = 100 µm.

The expression of SOX2 was evaluated in association with POU5F1 to determine the effect of *POU5F1* deletion on the initiation of ICM lineage specification (Fig. 6). *POU5F1* knockout did not alter SOX2 expression in morula stage embryos (Fig. 6A,B). The ratio of SOX2 positive cells to total cell number was not different between TKO and control morulas (Fig. 6C). The number of SOX2 positive cells was also not different between POU5F1 positive and negative embryos within the TKO group (Fig. 6D).

### *In-vitro* fertilized POU5F1 knockout embryos do not form expanded blastocysts

**Figure 7:**
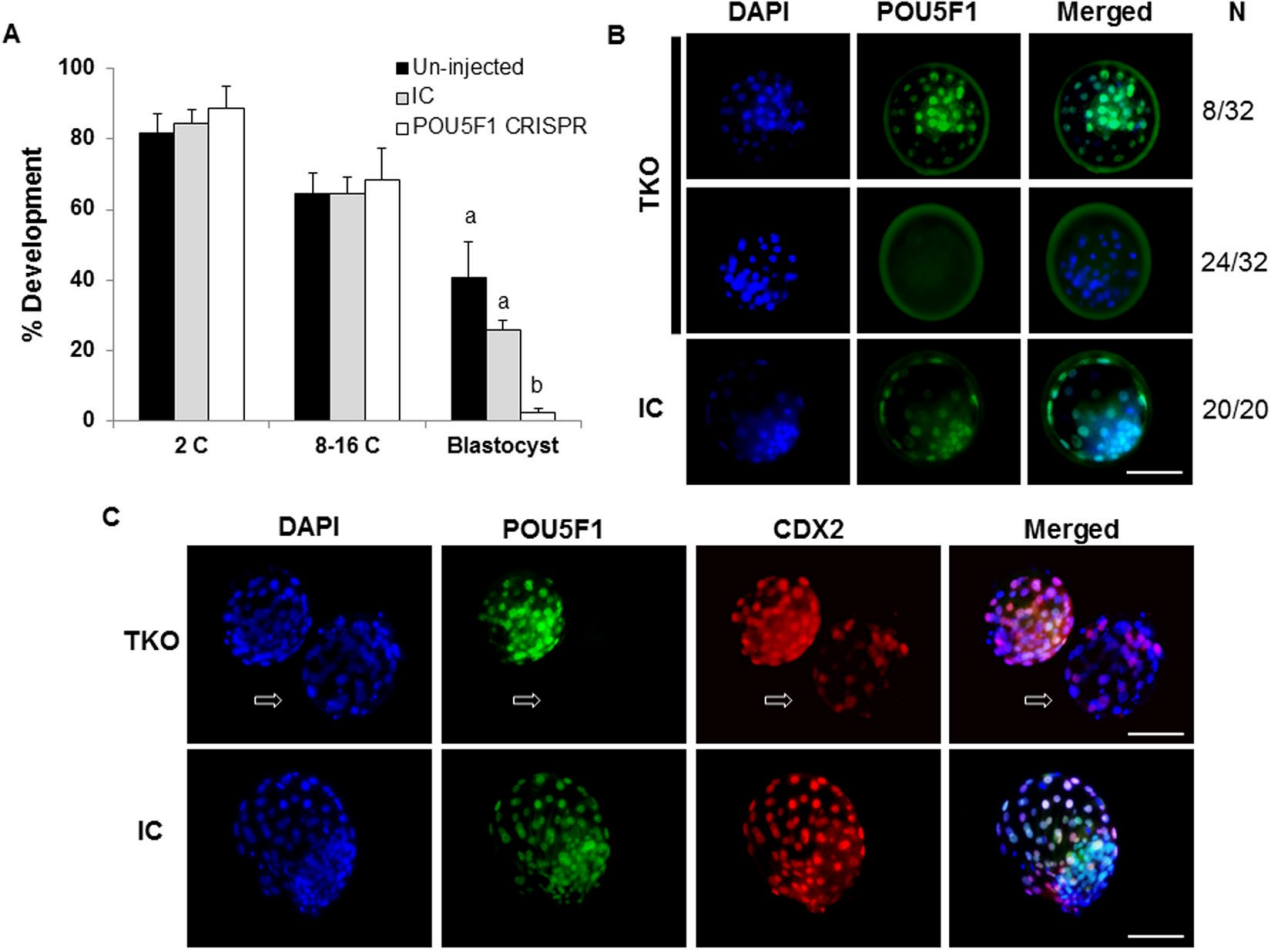
Zygotic POU5F1 is required for expanded blastocyst formation in *in vitro* fertilized bovine embryos. (**A**) Embryo development following peri-zygotic injection of CRISPR/Cas9 targeting the *POU5F1* locus was determined at the 2C (cell), 8–16C and blastocyst stages (D7.5) of oocytes fertilized with frozen-thawed bull sperm. (**B**) Immunofluorescent detection of POU5F1 expression was determined in D7.5 morulae from the targeted knockout (TKO) and injected control (IC) groups. (**C**) Developmentally advanced embryos from the TKO and IC groups were evaluated for POU5F1 (green) and CDX2 (red) expression. Arrows indicate a POU5F1 negative morula. ^a,b^Different letters indicate significant differences (P < 0.05). Scale bar = 100 µm.

*In-vitro* fertilized embryos were produced to determine the requirement of *POU5F1* for expanded blastocyst formation (Fig. 7). No differences were observed in developmental competence at the 2 or 8–16-cell stages. However, blastocyst formation in *POU5F1* TKO embryos was significantly lower (2.5%) than the injected and un-injected controls (26 and 41%, respectively). No expanded blastocysts were observed in the TKO group. CDX2 expression was apparent in D7.5 advanced morulas in both WT and *POU5F1* KO embryos, albeit with decreased or attenuated staining as observed in D7.5 parthenotes (Fig. 4). Depletion of *POU5F1* in D7.5 TKO morulas reached 75% (n = 32), whereas *POU5F1* was detected in 100% of all injected control embryos (n = 20). Embryos that stained positive for *POU5F1* staining from un-injected, injected control and TKO groups were confirmed to have wild-type genotype. *POU5F1* negative embryos (n = 3) from the TKO group that were sequenced confirmed the precense of mutations at the targeted site (Supplementary Table S1).

## Discussion

The functional genomics of bovine embryo development are largely undefined due to historic inefficiencies in gene editing and the inherently long generational gap associated with most large animal models appropriate for biomedical and agricultural research. Adaptation of the CRISPR/Cas9 system for directed intracytoplasmic injection of ribo-nucleoprotein constructs using a single sgRNA in a system devoid of cell lines and reconstructed embryos has allowed us to evaluate the function of bovine POU5F1 by targeted inactivation of the *POU5F1* gene with high efficiency and repeatability. Our results indicate the requirement of POU5F1 for bovine blastocyst formation, similar to recent findings in CRISPR-edited human embryos^19^. Following targeted disruption of *POU5F1*, we did not observe a negative effect on development to the 8–16C stage, which would be expected, since major embryonic POU5F1 expression occurs at the 8–16C stage^6,8,21^. In addition, no effect was observed on the ability of morulas to progress post-EGA up to D5, further suggesting that POU5F1 is not required for cell proliferation immediately after EGA. However, although some POU5F1^−/−^ embryos reached advanced morula stages, no expanded blastocysts were observed in the complete absence of POU5F1 in parthenogenetic and fertilized embryos, indicating a requirement for POU5F1 in orchestrating blastocyst formation and cell lineage specification beyond D5. Taken as a whole, the biological functions of POU5F1 are not required for bovine morula development but are evident for normal blastocyst formation as illustrated by lack of a defined ICM and failed expansion in TKO embryos. Our findings for the requirement of POU5F1 differ in comparison to SCNT-derived POU5F1 deficient bovine embryos in which the POU5F1 gene was mutated in fibroblast cells and embryos reconstructed following SCNT^22^. In SCNT-derived POU5F1 knockout embryos, POU5F1 was detected by immunofluorescence at the 8-cell and up to blastocyst stages with expression attributed to maternal stores of POU5F1^22^. Under our conditions, we could not detect POU5F1 in knockout embryos at any stage of development, maybe suggesting differences between fertilized and SCNT derived embryos.

In light of the phenotype observed with bovine POU5F1 ablation, the specific roles of POU5F1 for orchestrating embryo development appear to be distinct between species, since POU5F1 is not required for murine blastocyst development. POU5F1-deficient mice illustrate developmental roles for POU5F1 that are first detected during the second cell lineage specification, after blastocyst development^11^. POU5F1- null mice are able to produce blastocysts, albeit with alterations in epiblast (EPI) and primitive endoderm (PE) gene expression that point to a role of murine POU5F1 in second cell lineage specification and extraembryonic cell lineage determination^11^. In contrast, bovine embryos lacking POU5F1 undergo developmental arrest during the first lineage specification around embryonic D5, preventing blastocyst formation. Differences in cell lineage formation between mice and human embryos are further disparate at the blastocyst stage where factors localized to the trophectoderm lineage in mice are either not present or expressed in other cell lineages in human embryos^23^. In addition, requirements for cell signaling in EPI and PE are different between human and mouse^23^. Our observations in cattle are in close alignment with human POU5F1-null embryos^19^ in which blastocyst development was compromised following POU5F1 CRISPR genome editing. Despite a significant reduction in cattle blastocyst development, some embryos from the POU5F1 TKO group (n = 14) did develop to the blastocyst stage but were exclusively POU5F1 positive and un-edited as determined upon immuno-cytological and genotype evaluation. These observations coincide with the lack of consistent 100% mutation efficiency following CRISPR/Cas9 delivery.

Ablation of POU5F1 hindered the ability of bovine embryos to develop beyond the morula stage and the first cell lineage specification, similar to findings reported in human embryos^19^. At day 7.5, arrested morulas in control groups stained positive for POU5F1. On the other hand, 60% of arrested morulas were POU5F1 negative in the TKO group, which indicates successful mutation of the POU5F1 gene. In addition, the mutation efficiency of D7.5 sequenced morulas in TKO embryos was high (75%), and equally matched (75%) by immunofluorescent staining in IVF-produced embryos. The lack of blastocysts with absent POU5F1 staining supported by the high proportion of arrested morulas that lacked POU5F1, indicates that POU5F1 is necessary for blastocyst formation and in its absence, embryos arrest at the morula stage.

Zygotes targeted for *POU5F1* deletion did not display altered development prior to the 8–16C stage (D3) but consistently arrested at the morula stage when observed at the termination of culture (D7.5). Consistent with D7.5 TKO morulas, D5 TKO morulas also had a significant reduction in POU5F1 detection (36%), suggesting a high editing efficiency consistent with morula arrest and reduced blastocyst formation. Furthermore, some morula embryos showed mosaic POU5F1, consistent with expectations when editing zygotes due to the inability to control gene editing prior to DNA replication in all embryos^19^. Importantly, no POU5F1 mosaic blastocysts were found, indicating the requirement for ubiquitous expression or a threshold level of POU5F1 necessary for blastocyst development. While the potential for POU5F1 chimeric blastocysts in embryos targeted for deletion cannot be completely ruled out, all blastocysts from the TKO group contained >75% of their blastomeres staining positive for POU5F1, which was similar to the staining pattern observed in injected control blastocysts.

Morula cell number and development rate achieved by D5 was independent of POU5F1 expression, as shown when comparing total cell number between TKO and control groups or between POU5F1 positive (mosaic) and negative embryos within the TKO group. Equivalent cell numbers between groups indicate that lack of POU5F1 does not alter cell proliferation, and therefore the incapacity for blastocyst formation must be related to alterations in cell differentiation and not cell proliferation capacity that additionally, are not supported by mosaic expression of POU5F1.

SOX2 is a well-known marker of ICM development and as a POU5F1 binding partner^24,25^. Less is known about the requirement of POU5F1 for orchestrating ICM formation in the bovine embryo. Disruption of *POU5F1* prevented development beyond the morula stage but did not alter immunostaining detection of SOX2. The ability to detect SOX2 in POU5F1-KO embryos suggests the initiation of ICM formation followed shortly by developmental arrest. These findings are in close agreement with human embryos in which mutations affecting POU5F1 result in poor ICM formation followed by collapsed embryos^19^, pointing to a conserved role of human and cattle POU5F1 in preimplantation embryo development. Thus, a primary role of POU5F1 at the initiation of genome activation may be more related to maintenance rather than transcriptional regulation required for initial establishment of the inner-cell mass^11^. Notably, due to differences in the methods used to evaluate single cells from human embryos^19^ versus evaluation of whole cattle embryos herein as well as a reduced number of human embryos evaluated, direct comparisons of POU5F1 on embryo development suggest a number of conserved functions but have not been thoroughly investigated under identical conditions.

Blastocyst formation is dependent on formation of a functional TE. Interestingly, in arrested D7.5 morulas from the TKO group, CDX2 expression was altered. In bovine embryos POU5F1 is expressed in both ICM and TE lineages. Our results suggest and important role for POU5F1 in establishment of the TE lineage, since TE formation appeared to be compromised in POU5F1-KO embryos. The expression of CDX2 was evaluated in KO embryos as a marker of trophectoderm cell lineage specification first detected in advanced morula and blastocyst stages of development^8,26–28^. Day 7.5 blastocysts displayed normal CDX2 staining^27^. Likewise, all (18/18) D7.5 control morulas also expressed CDX2, albeit with attenuated staining intensity when compared to blastocysts. However, D7.5 POU5F1 KO morulas showed reduced and uncharacteristic CDX2 staining, while unedited embryos from the TKO group displayed similar CDX2 staining to the injected controls. POU5F1-null cells from human embryos show downregulation of CDX2^19^. In addition, lower levels of CDX2 transcripts have been reported in bovine POU5F1 knockdown embryos^9^. However, CDX2 transcripts are upregulated in POU5F1-deficient mouse embryos^11^. Taken together, disruption of the bovine *POU5F1* gene appears to result in failed trophectoderm specification as evidenced by lack of blastocyst formation and aberrant CDX2 expression.

In conclusion, direct intracytoplasmic zygotic injection of CRISPR/Cas9 using a single sgRNA was highly efficient at inducing *POU5F1* mutations and thus represents a valuable tool for investigating functional genomics of the bovine embryo. CRISPR-induced *POU5F1* mutations in bovine embryos resulted in developmental arrest prior to blastocyst formation but showed no alteration in development up to the morula stage. Consistent with the lack of blastocyst formation in POU5F1 KO embryos, CDX2, a TE marker, was aberrantly expressed in D7.5 embryos. On the other hand, SOX2, a pluripotency related gene that is specific to the ICM, was unaltered by lack of POU5F1 expression. Overall, bovine embryonic POU5F1 is required for expanded bovine blastocyst formation, and given the similarities observed in human POU5F1 KO embryos, the bovine embryos represents an excellent model for early human development.

## Methods

### Ethics Statement

No live animals were used in this research. Unless otherwise specified, reagents were purchased from Sigma-Aldrich, St. Louis, Missouri. USA.

### Embryo production

Bovine ovaries were obtained from a local abattoir. Follicles ranging between 3–6 mm in diameter were aspirated using a 20 gauge needle and syringe. Morphologically normal oocytes with a relatively uniform radial distribution of compact cumulus cells were selected and cultured for 22 h in Medium-199 with Earle salts (base medium) supplemented with 20 mM Na-pyruvate, 1 IU/ml FSH, 5 IU/ml LH (Sioux Biochemical), 1 µg/ml estradiol-17β, 10% fetal bovine serum (FBS, Hyclone, Logan, UT), and 0.1% gentamycin at 38.5 °C and 5% CO_2_ in humidity saturated air^29^. Expanded cumulus-oocyte-complexes (COCs) were vortexed for 4 min in 1% hyaluronidase and held in HEPES-based embryo culture medium (HH)^30^ for selection. Oocytes appearing morphologically normal with a homogeneously granulated cytoplasm and visible polar body were parthenogenetically activated by placement in a droplet of 5 µM ionomycin in HH for 4 min, washed three times in HH medium and placed in 2 mM of 6-DMAP in culture medium for 4 h to prevent second polar body extrusion^30^. Activated oocytes were washed five times and presumptive embryos were incubated in KSOM culture medium (EMD Millipore, Billerica, MA) supplemented with 0.3% BSA at 38.5 °C and 5% CO_2_ in air. The culture medium was supplemented with 5% FBS at 72 h post-activation (HPA). Following activation (D0), embryos were evaluated for cleavage at 48 h (D2), 8–16 cell stage at 72 h (D3) and for blastocyst development at 180 h (D7.5).

*In-vitro fertilized* embryos were collected and matured as reported herein. Following maturation, frozen-thawed sperm were passed through a 45:90% Percoll step gradient consisting of a HEPES buffered Tyrode’s lactate sperm medium (99 mM NaCl, 24.8 mM NaHCO_3_, 10 mM HEPES, 0.33 mM NaH_2_PO_4_, 24 mM sodium lactate (60%), 2.4 mM MgCl_2_ ∙ 2H_2_O, 2.6 mM CaCl_2_2H_2_O). COCs were co-incubated with motile sperm at a ratio of 8000:1 sperm per oocyte for 10 h in fertilization medium (114 mM NaCl, 25 mM NaHCO_3_, 3.2 mM KCl, 0.39 mM NaH_2_PO_4_, 0.18 mM penicillin-G, 16.6 mM sodium lactate, 0.76 mM MgCl_2_2H_2_O, 2.7 mM CaCl_2_2H_2_O) at 38.5 °C in 5% CO^2^ and humidity saturated air. COCs were then stripped of cumulus and CRISPR/CAS9 components were injected into presumptive zygotes as described above. Injected zygotes were then returned to KSOM culture medium and supplemented with 5% FBS at 72 HPI and cultured until D7.5 as described for activated embryos.

### CRISPR design and embryo microinjection

Guide RNA (gRNA) sequences were designed using AddGene Cas-designer software (rgenome.net/cas-designer) targeting the coding sequence of bovine POU5F1 at exons 1 and 2. The selected sequences were blasted against the bovine genome (blast.ncbi.nlm.nih.gov) to eliminate the potential for predicted off-target sites. The gRNA targeting exon 1 of *POU5F1* was determined to be ineffective for introducing mutations and was thus used as a control. Embryo micromanipulations were performed on a Nikon-Eclipse TE2000-U inverted microscope using an Origio ICSI needle with a 5.5 µm inner diameter and a custom holding pipette controlled by Vizio oil and Cell-Tram air microinjectors^30^. One-cell embryos were placed in 50 µl droplets of HH medium containing 10% FBS under oil and microinjections were performed at room temperature within 1 h of activation^10^. Synthetic gRNAs (Synthego) and Cas9 protein (PNA Bio) were delivered via intracytoplasmic injection at a concentration of 140 and 70 ng/μl, respectively. Following injections, lysed embryos were discarded and the remainder were placed in 50 µl droplets of culture medium and returned to incubation.

### DNA preparation and genotyping of single embryos

Single embryos ranging between 8–16 cell to blastocyst stage were individually collected in 10 μl of DNA lysis buffer (Epicentre Quick X-Tract), placed in a thermocycler at 65 °C for 6 min and 98 °C for 2 min^31^ and stored at −20 °C until use. Amplification of DNA was performed by nested PCR using primers (IDT, Table 1) spanning exon 2 on the bovine *POU5F1* locus. The first reaction consisted of a total volume of 20 µl (GoTaq Hot Start Green Master Mix – Promega). Five microliters of the first reaction were used as template for the nested reaction. PCR conditions were as follows: 95° for 3 min, followed by 35 cycles of 95° for 30 sec, 56° for 30 sec, 72° for 30 sec and a final extension of 72° for 7 min.

**Table 1:** Primer and sgRNA sequences (NCBI: AC_000180.1).

Primers	*POU5F1*	Sense		Length (nt)
Primers (Outer):	Exon 1	F	GTTGATCCTCGGACCTGGAT	1077
		R	AGCGGGTTAGATGCAGAAGA	
	Exon 2	F	CGTGTGTTTGTGAATGTGCG	1242
		R	GGAAAGAAATGGGCAGGCAA	
Primers (Inner):	Exon 1	F	CGCCCTATGACTTGTGTGG	858
		R	CGAAATTGGTGTTCCAGCTT	
	Exon 2	F	AGAGGGGGTGAGGTGGATAG	854
		R	CCAGTATCAGGGGGACAATG	
sgRNA	Exon 1	R	GCUUCUCCUUGUCCAGCUUC	
sgRNA	Exon 2	F	GAUCACACUAGGAUAUACCC	

Nested PCR reactions were visualized by gel electrophoresis on a 1% agarose gel with 0.1% ethidium bromide and run at 95 volts for 45 min followed by 2 ms UV exposure. PCR products resulting from individual embryos with a single band were purified using QIAquick Purification Kit (QIAGEN). Reactions containing more than one band were excised from agarose and gel-purified using QIAquick Gel extraction kit (QUIAGEN). Resulting amplicons from single embryos were submitted for Sanger sequencing to Quintara Biosciences (http://www.quintarabio.com/services, Allston, Ma). Sequences were analyzed using TIDE Analysis Software (https://tide-calcualt.nki.nl) for quantitative assessment of genome editing by sequence trace decomposition^32^. SnapGene software was used in complement with TIDE software to align sequences for characterization of mutation frequency and allele editing.

### Immunofluorescence staining

Embryos evaluated for immunofluorescence were fixed in 4% paraformaldehyde and held in PBS (0.1% PVA) under oil at 5 °C until evaluation. Fixed embryos were washed 3 × 10 min in PBS (0.1% Triton X-100) and then permeabilized for 30 min in PBS and 1% Triton X-100. Following a 10 min wash, embryos were placed in blocking buffer of PBS containing 0.1% Triton-X-100 with 1% BSA and 10% Normal Donkey Serum (sc-2044). Embryos were washed and then incubated overnight at 4 °C in antibody buffer (PBS with 0.1% Triton-X-100 and 1% BSA) at a 1:300 primary antibody concentration. All secondary staining was performed at a 1:500 dilution. POU5F1 staining was conducted using a goat polyclonal antibody (sc-8628) and detected by donkey anti-goat IgG-CFL 488 secondary staining (sc-362255). A CDX2 rabbit monoclonal antibody (AB7648) and SOX2 anti-rabbit (Biogenex AN833-RTU) followed by secondary staining with donkey, anti-rabbit 568 fluorophore (AB175470) were used to differentiate trophectoderm and inner cell mass, respectively. For co-immunostaining, identical primary and secondary concentrations were maintained in 80 µl total volumes. Embryos were mounted on a glass slide with a coverslip using Vector mounting solution containing DAPI for nuclear counterstaining and then sealed. Mounted embryos were imaged by epifluorescence microscopy with the following filter combinations and exposure times: donkey anti-rabbit 568- HCRed 41043 filter, Ex 575/50 –Em 640/50, 300 ms exposure time; donkey anti-goat 488- HQ:Y GFP 96345 filter, Ex 500/20 –Em 535/30, 2 s exposure; DAPI Vectashield-11000v3-UV filter, Ex 350/50–Em 420/lpv2, 4 ms exposure. Images were captured using Axiocam software and cells were objectively quantified using ImageJ software. Classification of D5 embryo knockout phenotype was achieved by standardization to injected control embryos following quantification of whole embryo POU5F1 blastomere expression as a percentage of total cell number by the following: Embryos expressing ≥75% POU5F1 were considered wild-type (+/+, WT), 1–74% mosaic (+/−) and 0% POU5F1 were complete (−/−) knockouts (KO). Analyses of targeted knockout (TKO) embryos included all phenotypes (+/+, +/−, −/−) from the TKO group. POU5F1 background staining was not removed from images of KO embryos but was linearly reduced in POU5F1 positive embryos to clearly distinguish characteristic nuclear staining.

### Statistical analyses

Developmental comparisons were calculated using a one-way ANOVA with Tukey’s-post hoc analysis to adjust for pairwise comparisons. Chi-squared tests were employed for analyses of categorical indel mutation type and t-tests were used to determine differences in cell number. Comparisons for cell number data excluded wild-type embryos in the TKO group and mutated embryos in the IC group. Embryo was considered the experimental unit for cell number data and replicate was the experimental unit for development rate. Fixed effects included treatment and random effect were assigned for replicate. Differences among treatments were considered significant when P < 0.05. Percentage data were assessed for normality (Shapiro-Wilk) and when necessary, arcsine transformed. SAS 9.0 software (St. Louis, Missouri) was used for all statistical analyses.

## Electronic supplementary material

Supplementary Table 1
