# Supplementary material for: Embryonic POU5F1 is Required for Expanded Bovine Blastocyst Formation
Source: Sci Rep. 2018 May 17;8:7753. doi: 10.1038/s41598-018-25964-x (PMC5958112; doi:10.1038/s41598-018-25964-x)
Supplement: Supplementary file 1 — Supplementary Table 1 [file 41598_2018_25964_MOESM1_ESM.pdf]

Supplementary Information

Title: Embryonic POU5F1 is Required for Expanded Bovine Blastocyst Formation

Bradford W. Daigneault[1‡], Sandeep Rajput[1], George W. Smith[1], Pablo J. Ross[2*]

Supplementary Table S1. Phenotype and genotype evaluation of IVF-produced embryos

	Immunofluorescence		Sequence	
Experimental group	POU5F1 +	POU5F1 -	Genotype	Total N
Un-injected Ctrl	8	0	WT	8
POU5F1 E1 Ctrl	3	0	WT	3
POU5F1 TKO	--	3	Mutated	3
POU5F1 TKO	2	--	WT	2

Day 7.5 embryos produced from *in vitro* fertilization with frozen-thawed bull sperm were fixed with 4% paraformaldehyde and subjected to immunohistochemistry to detect the presence or absence of POU5F1. A subset of embryos from control and targeted-knockout groups was sequenced to confirm that the genotype was consistent with POU5F1 expression.
